# Emergency Medicine Assistants in the Field of Toxicology, Comparison of ChatGPT-3.5 and GEMINI Artificial Intelligence Systems

**DOI:** 10.15388/Amed.2024.31.2.18

**Published:** 2024-12-04

**Authors:** Hatice Aslı Bedel, Cihan Bedel, Fatih Selvi, Ökkeş Zortuk, Yusuf Karancı

**Affiliations:** 1 Department of Pharmacology, Suleyman Demirel University, Faculty of Pharmacy, Isparta, Turkey; 2 Department of Emergency Medicine, Health Science University Antalya Training and Research Hospital, Antalya,Turkey; 3 Department of Emergency Medicine, Hatay Defne State Hospital, Hatay, Turkey

**Keywords:** ChatGPT, Gemini, emergency medicine, Raktažodžiai: ChatGPT, Gemini, skubioji medicina

## Abstract

**Objective:**

Artificial intelligence models human thinking and problem-solving abilities, allowing computers to make autonomous decisions. There is a lack of studies demonstrating the clinical utility of GPT and Gemin in the field of toxicology, which means their level of competence is not well understood. This study compares the responses given by GPT-3.5 and Gemin to those provided by emergency medicine residents.

**Methods:**

This prospective study was focused on toxicology and utilized the widely recognized educational resource ‘Tintinalli Emergency Medicine: A Comprehensive Study Guide’ for the field of Emergency Medicine. A set of twenty questions, each with five options, was devised to test knowledge of toxicological data as defined in the book. These questions were then used to train ChatGPT GPT-3.5 (Generative Pre-trained Transformer 3.5) by OpenAI and Gemini by Google AI in the clinic. The resulting answers were then meticulously analyzed.

**Results:**

28 physicians, 35.7% of whom were women, were included in our study. A comparison was made between the physician and AI scores. While a significant difference was found in the comparison (F=2.368 and p<0.001), no significant difference was found between the two groups in the post-hoc Tukey test. GPT-3.5 mean score is 9.9±0.71, Gemini mean score is 11.30±1.17 and, physicians’ mean score is 9.82±3.70 (Figure 1).

**Conclusions:**

It is clear that GPT-3.5 and Gemini respond similarly to topics in toxicology, just as resident physicians do.

## Abstract (it)

**Tikslas:**

Dirbtinis intelektas (DI) modeliuoja žmogaus mąstymą ir problemų sprendimo gebėjimus, todėl kompiuteriai gali savarankiškai priimti sprendimus. Tyrimų, įrodančių GPT ir Gemini klinikinį naudingumą toksikologijos srityje, trūksta, o tai reiškia, kad jų kompetencijos lygis nėra gerai žinomas. Šiame tyrime lyginami GPT-3.5 ir Gemini atsakymai su skubiosios medicinos rezidentų pateiktais atsakymais.

**Metodai:**

Šis prospektyvinis tyrimas buvo skirtas toksikologijai ir jame buvo naudojamas plačiai pripažintas mokomasis šaltinis „Tintinalli Emergency Medicine: A Comprehensive Study Guide“, skirtas skubiosios medicinos sričiai. Buvo sudarytas dvidešimties klausimų, kurių kiekvienas turėjo penkis atsakymų variantus, rinkinys, skirtas patikrinti knygoje apibrėžtų toksikologinių duomenų žinias. Vėliau šie klausimai buvo naudojami „ChatGPT GPT-3.5“ (Generative Pre-trained Transformer 3.5) pagal „OpenAI“ ir „Gemini“ pagal „Google AI“ klinikoje apmokyti „ChatGPT GPT-3.5“ (Generative Pre-trained Transformer 3.5). Tada gauti atsakymai buvo kruopščiai išanalizuoti.

**Rezultatai:**

Mūsų tyrime dalyvavo 28 gydytojai, iš kurių 35,7 proc. buvo moterys. Buvo palyginti gydytojų ir DI balai. Nors palyginimo metu nustatytas reikšmingas skirtumas (F = 2,368 ir p < 0,001), atlikus posthoc Tukey testą reikšmingo skirtumo tarp abiejų grupių nenustatyta. GPT-3.5 vidutinis balas yra 9,9±0,71, Gemini vidutinis balas – 11,30±1,17, o gydytojų vidutinis balas – 9,82±3,70 (1 pav.).

**Išvados:**

Akivaizdu, kad GPT-3.5 ir „Gemini“ panašiai atsako į toksikologijos temas, kaip ir gydytojai rezidentai.

## Introduction

Artificial Intelligence (AI) is a scientific discipline that has been around for nearly half a century. [1] It models human thinking and problem-solving abilities, allowing computers to make autonomous decisions. AI technologies encompass a range of complex tasks, including automation, robotics, image processing, language processing, and game development. They have the capability to learn and improve. [1,2] The progress in technology represents a significant potential for the future. It will accelerate transformations across many industries and sectors. In this context, applications such as Gemini and ChatGPT have been introduced into various fields. [3] While there have been many studies conducted with ChatGPT in the field of medicine in recent years, the accuracy rates have shown considerable variation, indicating that the topic is not yet fully clarified. The initially released and revolutionary ChatGPT GPT-3 model has since evolved into ChatGPT GPT-4. [4] Many studies have undertaken comprehensive comparisons between Gemini and ChatGPT to evaluate their performance. It is crucial to note that many of these studies have reached a pivotal point by comparing these methods using standardised measures. [5]

Clinical and Medical Toxicology is a branch of science that deals with the evaluation, diagnosis, and treatment of patients poisoned by drugs, chemicals, biological agents, and various other substances. [6] Toxicology remains a significant topic in both medical and pharmaceutical sciences due to its impact on increased mortality and morbidity. There is a clear need for more studies on the use of emerging technologies like Gemini and ChatGPT GPT in this field. [6,7] While the effectiveness of these technological solutions in addressing toxicology has not yet been fully determined, previous studies have indicated that these AI tools can be helpful in the diagnosis and clinical aspects of medical practice. [8] However, there are not enough studies demonstrating the clinical utility of ChatGPT GPT and Gemini in the field of toxicology, which means their level of competence is not well understood. Therefore, this study compares the responses given by ChatGPT GPT-3.5 and Gemini to those provided by emergency medicine residents.

## Material and Method

The study was conducted on residents who received training at the Emergency Medicine Clinic with permission from the Non-Interventional Scientific Studies Ethics Committee of SBU Antalya Training and Research Hospital. The test in question was administered to emergency service workers between the dates of 1 April 2024 and 1 October 2024. The study focused on toxicology and utilized the widely recognized educational resource ‘Tintinalli Emergency Medicine: A Comprehensive Study Guide’ for the field of Emergency Medicine. [9] A set of twenty questions, each with five options, was devised to test knowledge of toxicological data as defined in the book. These questions were then used to train ChatGPT GPT-3.5 (Generative Pre-trained Transformer 3.5) by OpenAI and Gemini by Google AI (2003) in the clinic. [10,11] The resulting answers were then meticulously analyzed.

## AI Answering

In the second phase of the study, we questioned two distinct artificial intelligence programs (ChatGPT GPT 3.5 and Gemini) and recorded their responses. Over a period of 20 days, we changed the order of the questions and asked the programs to answer them again. We then analyzed the responses with confidence.

## Sample Size

The G Power program has determined that a sample size of 12 participants per group, resulting in a total of 24 participants, is necessary with a significance level of 0.05, power of 0.99, and effect size of 1.75. [12]

## Statistical analysis

The study data was processed into a database and analysed using SPSS V.22 (IBM) for categorical data and normality distribution of numerical data. Graphs were drawn using GraphPad Prism 8 (Graphpad, Boston). Mean and standard deviation were used for data complying with normal distribution. Chi-square tests were performed for categorical data and Monte Carlo Exact was applied where relevant. The numerical data was divided into groups and an ANOVA test was conducted, followed by a post-hoc Tukey test. We considered only the data with a p-value below 0.05 as significant.

## Results

**Figure 1:**
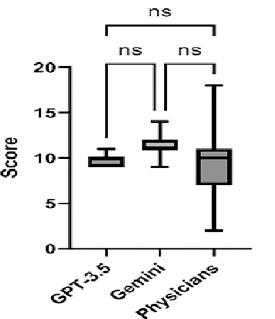
Comperison of answer scores

The study cohort comprised 28 physicians, 35.7% of whom were women. The demographic data of the physicians are presented in Table 1. A total of 20 questions were posed to the 28 physicians and two artificial intelligences who participated in the study. A comparison of the correct responses to the questions is presented in Table 2. Each correct answer to the 20 questions asked in the study was scored as 1 point. A comparison was made between the physicians and AI scores. While a significant difference was found in the comparison (Kruskal–Wallis statistic: 11.94 and p<0.001), GPT-3.5 mean score is 9.9±0.71, Gemini mean score is 11.30±1.17, and physicians’ mean score is 9.82±3.70 (Figure 1). Although GPT-3.5 gave in average approximately 50% correct answers and Gemini gave 55% correct answers, there was no significant difference (p=0.646).

**Table 1:** Demographic descriptions of study population

Description	
**Age (Mean±SD)**	29.25±3.18
**Gender (Female) n (%)**	10 (35.7)
**Year (Resident) n (%)**	
**1**	9 (32.1)
**2**	2 (7.1)
**3**	4 (14.3)
**4**	11 (39.3)
**Specialist**	2 (7.1)
**Work time (Mean±SD)**	4.75±2.90

**Table 2:** Comparison of answers

Questions n (%)	GPT-3.5 (n=20)	Gemini (n=20)	Physicians (n=28)	p-Value
**1**	19 (9.,0)	2 (10.0)	20 (71.4)	<0.001
**2**	0	7 (35)	22 (78.6)	<0.001
**3**	3 (15)	0 (0)	10 (35.7)	0.007
**4**	20 (100)	20 (100)	15 (55.6)	<0.001
**5**	0	20 (100)	13 (46.4)	<0.001
**6**	0	14 (70)	10 (35.7)	<0.001
**7**	20 (100)	20 (100)	21 (75)	0.004
**8**	0	20 (100)	15 (53.6)	<0.001
**9**	20 (100)	2 (10)	13 (48.1)	<0.001
**10**	5 (25)	0	7 (25)	0.048
**11**	18 (90)	20 (100)	9 (32.1)	<0.001
**12**	9 (45)	4 (20)	14 (50)	0.095
**13**	18 (90)	20 (100)	11 (39.3)	<0.001
**14**	0	2 (10)	5 (17.9)	0.133
**15**	1 (5)	4 (20)	12 (44.4)	0.007
**16**	18 (90)	20 (100)	19 (67.9)	0.008
**17**	3 (15)	16 (80)	6 (21.4)	<0.001
**18**	18 (90)	15 (75)	18 (64.3)	0.128
**19**	6 (30)	0	13 (46.4)	0.002
**20**	20 (100)	20 (100)	22 (78,6)	0.006

## Discussion

Our study found no significant differences in responses provided by Gemini, ChatGPT and emergency medicine residents. Technological programs performed similarly to emergency medicine residents in answering questions. While there are studies demonstrating the appropriate use of ChatGPT in various international exams, a few studies have shown that it may lack sufficient reliability. ChatGPT is a member of the family, with previous versions being GPT-2 and GPT-3. [3-7] It is trained using advanced artificial intelligence technology, offering high accuracy and versatility. These models enable users to ask questions, generate written text, and perform many other tasks. [6-9]

The integration of Gemini and ChatGPT into application layers will undoubtedly result in the emergence of unexpected harmful behaviours that are difficult to trace back and, therefore, challenging to correct at their source. [13] Products are traditionally released with specification sheets detailing their limitations. ChatGPT and other GPT models are trained using large amounts of text data collected from the internet. This data is typically sourced from websites, articles, blog posts, social media content, and book chapters. [13,14] While AI technology may not yet fully achieve human-like features, it will undoubtedly improve significantly in the future. [15] Researchers leveraging this modern technology in the medical field can offer valuable insights into how it might affect and transform various domains. [16] ChatGPT’s ability to analyse clinical trial data and research articles makes it a valuable tool for identifying new drug targets and even aiding in the design of these targets based on their chemical and physical properties. This technological advancement will significantly impact medical research and development. [17] Our study found that the mean score for GPT-3.5 was 9.9±0.71, for Gemini was 11.30±1.17, and for physicians was 9.82±3.70.

ChatGPT and Gemini are models that may be useful for drug development. They can predict a molecule’s pharmacokinetic, pharmacodynamic, and toxicity properties, identifying new drug targets and even designing them based on their chemical and physical characteristics. They can also forecast a molecule’s pharmacokinetic, pharmacodynamic, and toxicity profiles, providing critical insights for drug development. [16-19] ChatGPT excels at identifying similar molecules with a higher potential for success in preclinical and clinical trials. It can also improve clinical trial design, assist in participant recruitment, and facilitate efficient and effective clinical trials by leveraging pattern recognition and analytical capabilities. [7] Moreover, both ChatGPT and Gemini have demonstrated vulnerabilities regarding their ability to replace human brain functions in medical materials. However, they can function effectively as virtual teaching assistants, providing comprehensive and relevant information to students. [7-10] They also have the potential to facilitate interactive simulations and improve learning techniques, ultimately advancing education. However, it is important to note that ChatGPT lacks the ability to reference sources independently, which could result in it inadvertently quoting from another source or offering misleading or biased responses. [16-20] This limitation is especially concerning when users rely on its suggestions for healthcare advice, accepting outcomes as “good enough.” Our study found no significant reliability between people and these programs when assessing responses. Additionally, both Gemini and ChatGPT-4 have limitations in terms of advanced critical thinking and problem-solving skills, which impact their usefulness in contexts where creative and critical application of information is essential, such as in health education. [21] These limitations raise concerns about AI’s reliability as a teaching tool. Given these challenges, it is crucial to comprehensively evaluate widely used AI chatbots like ChatGPT and Gemini in various educational contexts. [22] This assessment is essential for advancing AI algorithms to improve performance, which will ultimately lead to better outcomes across various disciplines, including health education. [23] AI models often struggle to understand the context and nuance that are crucial to critical thinking. They can process information, but they may not grasp the subtleties of human language and thought processes, leading to errors in judgment and decision-making. [20] Critical thinking includes empathy, ethics, and problem-solving, which AI cannot replicate with the same agility and standards as humans. These traits are essential for informed decision-making and are difficult to code into AI systems. [17-20] Over-reliance on AI tools can inhibit the development of independent problem-solving and critical thinking skills in humans. This reliance can lead to a decrease in human cognitive engagement and creativity. [19-22] Our study found no significant differences in the responses provided by Gemini, ChatGPT, and emergency medicine residents. The performance of these AI systems overlaps with the performance of emergency medical assistants and shows that they have a similar result in responding to medical questions.

The incorporation of artificial intelligence (AI) into clinical practice is becoming increasingly regarded as a means of providing support to healthcare professionals, rather than as a means of replacing them. [24] The potential of AI to enhance patient care, improve diagnostic accuracy and streamline workflows is well documented. However, the successful implementation of AI in healthcare requires careful consideration of both technological and human factors. [25] The objective should be to establish a symbiotic relationship in which AI tools enhance the capabilities of healthcare providers, thereby ensuring that patient care is both efficient and personalised. [26] This approach underscores the necessity of human supervision and expertise in clinical decision-making, while capitalising on the capabilities of AI in data analysis and pattern recognition. The application of AI algorithms has been shown to result in highly accurate interpretations of medical images, including X-rays and MRIs. This has the potential to significantly reduce the time required for diagnosis and improve early detection rates. [24,27] By analysing large datasets, AI is able to identify patterns and anomalies that may otherwise be overlooked by human observers, thereby supporting clinicians in making more informed decisions. [27] The capacity of AI to process vast quantities of patient data enables the creation of personalised treatment plans that take into account the specific characteristics, medical history and genetic information of each individual patient. [24,27,28] This data-driven approach has the potential to optimise patient care by reducing reliance on trial-and-error methods and tailoring interventions to the specific needs of each patient. [29,30]

The study we conducted has some limitations. The first of these is that it was designed as a single-centre study, which limits the generalisability of the results. Another significant constraint is that the individual education and knowledge levels of those providing the responses may vary, potentially impacting the consistency of the data. Although there might be variations in individual responses, it is clear that determining the importance of the topic will require involving broader populations with diverse learning methods to get a more comprehensive understanding. In order to understand the value of our study, further studies with larger populations, multicentre, more complex clinical scenarios should be planned.

## Conclusion

It is clear that GPT-3.5 and Gemini respond similarly to topics in toxicology, just as resident physicians do. Gemini and GPT have been shown to respond to toxicology issues in a similar way with an assistant.
